# First evaluation of the impact of a targeted subunit vaccine against bovine viral diarrhea virus in feedlot cattle

**DOI:** 10.1093/tas/txae046

**Published:** 2024-04-06

**Authors:** Demian Bellido, Emanuel R Gumina, Gonzalo J Rodríguez Senes, Franco M Chiariotti, Maximiliano Audrito, Pedro M Sueldo, Gustavo M Sueldo, Andrés Wigdorovitz

**Affiliations:** Vetanco SA, Chile 33, Villa Martelli, Buenos Aires, ArgentinaB1603CMA; Bioinnovo SA, Dr Nicolas Repetto y Los Reseros S/N, Hurlingham, Buenos Aires, ArgentinaB1681FUU; Vetanco SA, Chile 33, Villa Martelli, Buenos Aires, ArgentinaB1603CMA; Vetanco SA, Chile 33, Villa Martelli, Buenos Aires, ArgentinaB1603CMA; Vetanco SA, Chile 33, Villa Martelli, Buenos Aires, ArgentinaB1603CMA; Agro sin Fronteras, JJ Paso 452, Marcos Juarez, Córdoba, ArgentinaX2580DML; Vetanco SA, Chile 33, Villa Martelli, Buenos Aires, ArgentinaB1603CMA; Agro sin Fronteras, JJ Paso 452, Marcos Juarez, Córdoba, ArgentinaX2580DML; Vetanco SA, Chile 33, Villa Martelli, Buenos Aires, ArgentinaB1603CMA; Incuinta, IVIT INTA, Dr N. Repetto y Los Reseros S/N, Hurlingham, Buenos Aires, ArgentinaB1681FUU

**Keywords:** bovine viral diarrhea virus, bovine respiratory disease, feedlot cattle, field trial, subunit targeted vaccine

## Abstract

Bovine respiratory disease (BRD) is a serious health and economic problem in the beef industry, which is often associated with transportation and caused by different pathogens. In this study, we evaluated the effect of a novel subunit targeted vaccine against bovine viral diarrhea virus (BVDV) in feedlot cattle, a major viral agent of BRD. The core of this novel vaccine is the fusion of the BVDV structural glycoprotein, E2, to a single-chain antibody, APCH, together termed, APCH-E2. The APCH antibody targets the E2 antigen to the major histocompatibility type II molecule (MHC-II) present in antigen-presenting cells. To evaluate the vaccine, 2,992 animals were randomly allocated into two groups, control group (*N* = 1,491) and treatment group (*N* = 1,501). Animals of both groups received the routine sanitary plan: two doses of clostridial, respiratory, and rabies vaccines. Animals within the treatment group also received two doses of a targeted subunit vaccine against BVDV. Serum samples were taken on the day of the first inoculation (T0) and 90 d later (T90). Viral circulation was monitored using an anti-P80 ELISA (virus-specific) and immune response was evaluated by anti-E2 ELISA (detects virus and vaccine immune responses). Only animals treated for respiratory disease were considered positive cases of BRD. Results demonstrate that the control group had significantly more animals treated for BRD cases compared to the treatment group (5.9% vs. 3.7%, *P* = 0.02). The control group had a greater number of animals positive for anti-P80 antibodies and significantly fewer animals positive for anti-E2 antibodies compared to the treatment group (69% vs. 61% and 71% vs. 99%, respectively, *P* = 0.003), consistent with natural viral circulation within this group. The treatment group, conversely, had fewer animals positive for anti-P80 antibodies and a greater number of animals positive for anti-E2 antibodies, consistent with a robust vaccine-induced antibody response and a reduction of the BVDV circulation within this group. The data indicate the new subunit targeted vaccine induced greater anti-E2 antibodies and reduced the amount of BVD virus circulation within the treatment group leading to a fewer number of animals needing to be treated for BRD.

## Teaser

A novel targeted subunit vaccine against BVDV was able to reduce viral circulation and bovine respiratory disease cases in a trial with almost 3,000 animals in a commercial feedlot.

## Introduction

Bovine viral diarrhea virus (BVDV), is one of the most important bovine pathogens worldwide and is responsible for enormous production losses in beef and dairy herds (Houe, 1999; Gunn, et al., 2004; Gunn et al., 2005). BVDV belongs to the genus *Pestivirus* within the family *Flaviviridae* and includes three species, BVDV-1 (Pestivirus A), BVDV-2 (Pestivirus B), and Hobi-like pestivirus (HoBiPeV; Pestivirus H; ICTV, 2020). The genome consists of a single-stranded, positive-sense RNA of 12.3–13 kb encoding a single open reading frame, which is flanked by 5ʹ- and 3ʹ-untranslated regions (UTRs; ICTV, 2020). A trademark of the BDV viruses is immunosuppression that leads to a decrease in the number of white cells and platelets and a misfunction of immune cells in BVDV-infected cattle (Roth and Kaeberle, 1983; Walz et al., 2001; Kelling et al., 2002; Chase et al., 2015; Abdelsalam et al., 2020). It also leads to the occurrence of increased disease and pathology severity when BVDV-infected cattle are coinfected with other pathogens such as *Mannheimia haemolytica*, bovine herpesvirus-1, bovine respiratory syncytial virus, and bovine Coronavirus (Potgieter et al., 1984a, b; Kelling et al., 1996; Fulton et al., 2000; Burciaga-Robles et al., 2010; Carlos-Valdez et al., 2016; Ridpath et al., 2020). bovine respiratory disease (BRD) may cause the greatest economic impact on the cattle feeding industry (feedlot) because of increased health-related costs from morbidity and fatalities as well as decreased performance (Smith, 2000; Houe, 2003). The relationship between BVDV and BRD has been extensively reported (Bielefeldt-Ohmann, 1995; Hessman et al., 2009; Ridpath, 2010; Hay et al., 2016; Mitra et al., 2016). Serologic studies have demonstrated that feedlot animals with higher antibody titers via seroconversion from BVDV exposure or vaccination are at a lower risk of developing BRD (Walz et al., 2010; Grooms et al., 2014; Theurer, et al., 2015; van Oirschot, et al., no date). Additionally, a prospective cohort study by Booker (2002) of new cases of BRD occurring after day 70 of the feeding period (cases) and “healthy” pen mates (controls) demonstrated that BVDV was 4.55 times more likely to be isolated from the serum of case animals than control animals. Vaccination against BVDV is an important component of prevention and control programs since it can prevent clinical signs of BRD, reduce viral spread, and the birth of new persistently infected animals. In most countries, only modified live vaccines (MLV) and inactivated vaccines are used in vaccination programs. Both have historical disadvantages; MLV in terms of safety and inactivated vaccines in terms of immunoprotection. Over the last decade, our research group developed and optimized the first inactivated subunit BVDV vaccine. The core of the vaccine is the E2 protein (Singer strain, BVDV 1a) of the virus fused to a targeting molecule called APCH (Pecora et al., 2015). The APCH molecule is a single-chain antibody against an invariant MHC-II epitope. It was first developed and tested in swine but has now been shown to cross-react with several species, including bovines (Argilaguet et al., 2011; Borrego et al., 2011; Gil et al., 2011). The vaccine is produced in SF9 cells utilizing the baculovirus production system and was first released to the market in 2018 with results in guinea pigs and cattle previously published (Bellido et al., 2020). In the present work, we present the results obtained in a commercial feedlot yard with this novel vaccine. Immune response, viral circulation, and BRD cases were evaluated in animals vaccinated after the arrival to the feedlot yard. To the best of our knowledge, this is the first trial to evaluate the performance of a BVDV vaccine in a feedlot yard conducted in Argentina.

## Materials and Methods

“The research was carried out according to the Guide for the Care and Use of Laboratory Animals (NRC, 2011) as outlined by AAALAC International and the Guide for the Care and Use of Agricultural Animals in Research and Teaching (third ed. 2010) as outlined by The American Society of Animal Science (Fass, 2010).”

### Study Facilities

A feedlot in Córdoba, Argentina, was selected for feedlot calf enrollment. The capacity of this feedlot is 24,000 animals and more than 70,000 calves are fed annually. The basic design of the feedlot is representative of the standard design used in Argentina. The animals were housed in open-air, dirt-floor pens, arranged side-by-side, with central feed alleys and 20% porosity wood-fence windbreaks. Each pen holds approximately 150 to 200 animals.

Veterinary hospital and cattle handling facilities are located in the feedlot yard. Each cattle handling facility has a hydraulic chute, an electronic scale (accuracy range of 0.1 kg.), a chute-side computer for animal health data collection (Tru-Test model XR0500), and separation alleys to facilitate the return of animals to designated pens.

### Study Animals

The animals enrolled in this study (2,992 calves; 30 pens) ranged in age from 12 to 36 mo old and were crossbred heifers, steer, and cows of different breeds (Holstein, Braford, Brangus, Hereford, and Aberdeen Angus). The animals were purchased from auction markets throughout the Córdoba and Santa Fe provinces using the standard procurement procedures employed by the feedlot. Animals were transported by truck to the feedlots after assembly at the auction markets. Upon arrival at the feedlot, all animals were moved through a hydraulic chute for processing. At processing, animals of both groups received the following: unique individual animal identification tag; a combined vaccine containing inactivated cultures of infectious bovine rhinotracheitis virus, BVDV, parainfluenza-3 virus (PI3), and bovine syncytial respiratory virus and bacterins of *Mannheimia haemolytica*, *Pasteurella multocida,* and *Histophilus somni*; a multivalent clostridial bacterin/toxoid; and a topical external and internal parasite control product. Three weeks post-arrival animals received the booster dose of the abovementioned vaccines.

### Experimental Design

Animals were randomly divided into two groups, control group (*N* = 1,491) and treated group (*N* = 1,501). In addition to the standard processing procedures described above, animals in the treated group received a 3 mL dose of the subunit vaccine (Vedevax Block, Bioinnovo SA, Bs As, Argentina). Treated group animals also received a second 3 mL dose at the 3-wk vaccination booster interval described above. Serum samples (collected from the jugular vein) were collected from 5% of animals of both study groups at processing (day 0) and 90 d later (day 90). Viral circulation was monitored using an anti-P80 ELISA (virus-specific [González et al., 2014; Rosete Fernández et al., 2023]) and immune response was evaluated by anti-E2 ELISA (detects virus and vaccine immune responses). Only animals treated with medication for respiratory disease were considered positive cases of BRD.

### E2 and P80 ELISA

Immune response was evaluated by anti-E2 Competition ELISA as described previously by Bellido et al. (2020). Viral circulation was evaluated using a commercial anti-P80 ELISA (CIVTEST BOVIS BVD/BD P80, HIPRA) following the manufacturer’s instructions.

### Statistical Analysis

Immune response, viral circulation, and percent BRD positive (%BRD) data were analyzed using Fisher’s Exact Test (GraphPad Prism 9.0.2., GraphPad Software, San Diego, California, USA) comparing the absolute positive and negative samples between treatment groups for each sample time point establishing a statistical significance of *P* < 0.05. average daily gain (ADG) was analyzed using T Student for independent sample point establishing a statistical significance of *P* < 0.05.

## Results

To assess the impact of this novel vaccine on BRD, a feedlot trial was conducted in cattle. The field trial was conducted in a commercial feedlot under normal management conditions as mentioned in the introduction. BVDV was present on the farm selected for testing and therefore antibody titers against BVDV were observed at the start of the study using an anti-E2 ELISA. It is important to mention that this assay recognizes antibodies produced by both natural infection and vaccination.

**Figure 1.**
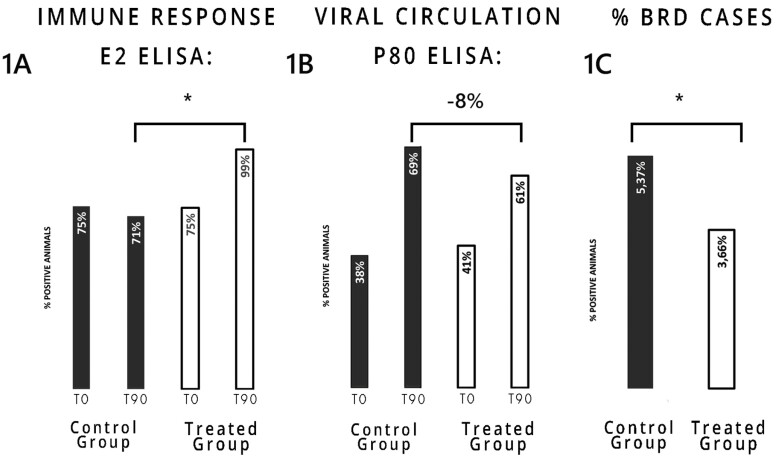
Full bars: control group; empty bars: treated group. (A) Percentage of antibody-positive animals to E2 protein on days 0 and 90 in each group. (B) Percentage of antibody-positive animals to P80 protein on days 0 and 90 in each group. (C) bovine respiratory disease cases in each group. * Indicates statistical significance (*P* ≤ 0.05).

At day 0, 75% of the animals of both groups were positive for anti-E2 antibodies. By day 90 of the study, significantly more cattle in the treated group had detectable anti-E2 antibodies as compared to the control group (99% vs. 71%, *P* = 0.003, Figure 1A).

To evaluate BVDV circulation in the feedlot yard an anti-P80 ELISA was used. This ELISA only recognizes antibodies against the nonstructural protein P80 present in the infected animals. It is important to mention that vaccinated animals do not present antibodies against P80(González et al., 2014; Rosete Fernández et al., 2023). Animals in both groups presented similar percentages of seropositivity at day 0, 38% in the control group and 41% in the treated group, but at day 90 69% of the animals in the control group were positive compared to 61% in the treated group (Figure 1B), indicating a 35% reduction in the number of infected animals in the treated group. Any animal that received treatment for BRD was considered a positive BRD case in this analysis. The control group had significantly more BRD cases compared to the treated group (5.9% vs. 3.7%, *P* = 0.02; Figure 1C), representing a 32% decrease in the number of animals needing treatment for BRD. There were no differences in animal weight gain and mortality during the trial among groups (data not shown). The analysis indicated control group cattle were 1.4 times more likely to be treated for BRD than treated group cattle (*P* = 0.02).

## Discussion

BRD is one of the major problems that threaten cattle in feedlot yards. It has an especially important sanitary and economic impact on the feedlot industry. Vaccination on arrival with combined inactivated or MLV for respiratory pathogens is currently the primary method with which feedlot operators attempt to prevent and control BRD cases. From an immunological point of view, animals should be vaccinated 15 to 30 d prior to feedlot entry to fully develop the immune response before encounter with BRD agents; however, for practical and economic reasons, most of the animals enter without a history of vaccination and receive the first dose within the first week of residence in the feedlot. Prophylactic use of antibiotics, although highly controversial and not recommended, is also used to prevent BRD cases in feedlot cattle. Historically, only MLVs or killed vaccines were available for the prevention of BVDV. The use of MLVs in cattle has several disadvantages. Primarily, the replication of the vaccine strains can lead to an initial reduction in daily weight gain (Richeson et al., 2008). Secondarily, certain vaccine strains, including BVDV and BoHV-1, have been shown to have the potential to cross into the placenta resulting in fetal infections (Fulton, 2009). Lastly, Fulton et al. found vaccine strains of BoHV-1, BVDV, and PI3V in nasal swabs of animals treated for BRD and in the lungs of necropsy animals who died from BRD ^32^. Taken together, MLVs still pose a risk to feedlot cattle. Furthermore, in Argentina MLV vaccines are forbidden, only killed or inactivated vaccines are allowed for use in livestock production. Inactivated vaccines have some issues in beef cattle since stress reduces the immune response induced by non-replicating vaccines diminishing the effectiveness of such vaccines in feedlot yards (Richeson, 2015). The objective of the present trial was to evaluate the effectiveness of a novel subunit-targeted vaccine against BVDV in a commercial feedlot yard. The vaccine was incorporated into the standard processing procedures and was administered simultaneously with a commercial respiratory vaccine which contains inactivated BVDV in its formulation. Three different parameters were measured to assess the effectiveness of the vaccine: (1) anti-E2 antibody response (detects virus and vaccine immune responses), E2 is the immunodominant protein of the virus and the viral antigen of the subunit targeted vaccine, (2) viral circulation, using anti-P80 ELISA since P80 is a nonstructural protein that works as infection-marker, and (3) BRD cases. The percentage of animals with detectable anti-E2 antibodies at trial D0 was equal to 75%. At day 90 of the trial, 99% of treated animals had detectable anti-E2 antibodies as compared to 71% in the control, indicating the sole BVDV vaccine received by the control animals is insufficient to produce a sustained anti-E2 serum antibody response. These results indicate a robust, sustained, and consistent immune response is induced by the addition of this novel vaccine to the treatment program despite the stressful conditions faced by the animals in the feedlot yard. A similar percentage level of anti-E2 antibodies was induced in a previous trial in which animals were immunized with the targeted vaccine alone, without the addition of an inactivated vaccine against BVDV. In that trial it was also shown the antibody level reached after immunization with the targeted vaccine was independent of the existing antibodies against BVDV (Bellido et al., 2020). However, a synergistic effect between the inactivated vaccine and the targeted vaccine cannot ruled out in this trial, but the data clearly indicated a positive effect on immunity when the targeted vaccine is utilized in a feedlot system. Viral circulation was diminished in the treated group. At day 0, both groups had similar anti-P80 antibody positivity rates, 38% for the control group and 41% for the treated group. At day 90, 69% (+ 31%) of the control animals and 61% (+ 20%) of the treated animals had detectable anti-P80 antibodies. Treatment for BRD cases were significantly reduced in the treated group compared to the control group (3.7% vs. 5.4%, *P* = 0.02). Statistical analysis indicates animals in the treated group have a 40% decrease in the relative risk of developing BRD. Correspondingly, the increase in E2 immune response and the decrease in viral circulation showed similar values, 39% and 35%, respectively. Although no differences were observed between the treated and control groups in terms of weight gain, a significant difference in ADG was observed between the sick animals, animals that were treated for BRD whether from the control or treated group, (ADG = 0.89 kg/sick animal) and the healthy animals (ADG = 1.23 kg/healthy animal). With a difference in 25 BRD cases between the treated and control groups, this equates to an additional weight gain of approximately 1,871 kg in the treated group compared to the control in this 90-d period. Furthermore, the treated cattle had fewer total animals that needed to be treated with antibiotics for any health issue compared to the control group (*P* = 0.0441), suggesting addition of the inactivated subunit vaccine to the standard care protocol may contribute to overall better health of the animal and limit BVDV-induced immunosuppression.

The addition of this recently available subunit vaccine into the standard vaccination program for growing animals induced a robust and long-lasting immune response against BVDV and a reduction in the risk of developing BRD. These results are in concordance with previous research that shows an association between high antibody titers against BVDV with greater protection from BRD (Moerman et al., 1994; O’Connor et al., 2001), therefore, producers should strongly consider designing or modifying standard treatment plans to induce the highest titers of anti-BVDV antibodies in their cattle as means to reduces cases of BRD and increase profitably. It also contributes to a reduction in antibiotic use, which benefits the farmer, the consumer, and the environment.
